# Can agricultural technological progress promote China’s interprovincial rural revitalization? an analytical perspective based on agricultural-scale operations

**DOI:** 10.1371/journal.pone.0309339

**Published:** 2024-12-06

**Authors:** Fei Wang, Dong Xue, Zheyi Yang

**Affiliations:** 1 School of Economics, Henan University, Kaifeng, China; 2 Zhengzhou Business University, Zhengzhou, China; 3 Central China Securities, Zhengzhou, China; Qufu Normal University, CHINA

## Abstract

To clarify the role of agricultural technological progress in the process of rural revitalization, this paper uses the agricultural panel data of 31 provinces in China from 2007 to 2020 to measure the Total Factor Productivity of agriculture, analyzes the impact direction and spatial spillover effect of agricultural technological progress on China’s rural revitalization through the spatial Durbin model, and analyzes the threshold mechanism of agricultural technological progress on China’s rural revitalization by using the panel threshold model. The results are as follows: (1) The spatial and geographical agglomeration of interprovincial rural revitalization in China has gradually weakened, and the regional imbalance has improved; (2) The progress in agricultural technology plays a positive role in promoting China’s interprovincial rural revitalization, and the overall nonlinear characteristics of "first inhibiting and then promoting" are presented, and the conclusion is still robust after fully considering the factors of time, region and economic distance; (3) Further analysis shows that the impact of agricultural technological progress on China’s interprovincial rural revitalization is based on the threshold constraints of land-scale operation and agricultural-industry agglomeration, and shows significant spatial heterogeneity. The inherent reason is that whether the land-scale operation entity adopts long-term investment decisions such as new agricultural technology depends on the expectation of land management risk stability, while agricultural-industry agglomeration hinders the diffusion and spillover of agricultural technology due to the exclusive characteristics of agricultural production geographical locations. Therefore, in the future of China’s rural revitalization, the government should guide the large-scale operation of land and agricultural production according to local conditions to give full play to the positive spillover effect and spatial radiation capacity of agricultural technology.

## Introduction

In recent years, due to the continuous advancement of urbanization and the prominent contradiction between urban and rural development, the Chinese government has launched and implemented a rural revitalization strategy to further promote rural development in the new era. In particular, the report of the 19th National Congress of the Communist Party of China clearly points out that the rural revitalization strategy is the general starting point for revitalizing agriculture and rural areas in the new era, indicating that China’s rural areas have entered a new period of development and transformation. Thus, in the transition period, China must build an agricultural modernization development system, improve the level of agricultural mechanization, promote the innovation and promotion of agricultural technology, practice green development, and steadily promote the implementation of the strategy of high-quality development of the rural economy to support rural agricultural development.

Rural revitalization requires the development of modern agriculture, so China is attaching more importance to and is relying more on scientific and technological progress in agriculture than ever before. China noted that the key to agricultural modernization lies in scientific and technological progress in agriculture. According to the neoclassical theory of economic growth, technological progress plays a decisive role in driving economic growth (Lucas, 1988) [1]. During the transition period of China’s rural revitalization, the progress in agricultural technology has attracted the attention of many domestic scholars, and much research has been conducted to promote China’s rural development and an important means to achieve success for rural industries (Guo et al. 2010; Li et al. 2011; Meng 2015; Traore et al. 2019; Tan et al. 2022) [2–6]. In this context, the contribution rate of China’s agricultural science and technology progress has also been greatly increased through relevant agricultural technology innovation and promotion measures, such as providing policy support, financial tilting, and training and guidance.

It is worth noting that although China is currently experiencing great changes in rural areas, the smallholder farmer production model is still the main type of agricultural production in China, and the cognition and adoption decisions of smallholder and large-scale operators are not the same; thus, there is a mechanism that restricts the progress of agricultural technology in the expansion of the agricultural operation scale (Zhao 2012; Xu and Liu 2013) [7,8]. Ju (2018), Xu et al. (2022) found that agricultural scale management, as one of the driving forces of agricultural technological progress, improves the utilization efficiency of agricultural production factors and strengthens the advantages of the agricultural industry by playing the role of an intermediary variable [9,10]. Based on the experience of China and that of other developing countries, scholars such as Wei et al. (2023) [11], Zhu et al. (2022) [12], Berbeka and Borodako (2018) pointed out that the expansion of the agricultural operation scale will promote the popularization and application of low-carbon and ecological technologies, change the use intensity of chemical fertilizers and machinery, and lead to and support the development of green and low-carbon agriculture, which means that the impact of agricultural progress and progress in China’s rural revitalization is obviously constrained by the scale of agricultural operations [11,13,14]. In addition, the open-air nature of agricultural production facilitates the spatial spillover of agricultural technology, and the existence of this externality enables adjacent areas to improve their own local rural revitalization through learning and imitation; however, the labor substitution generated by agricultural technological progress will inevitably lead to the transfer of production factors such as labor, so the impact of agricultural technological progress on China’s interprovincial rural revitalization may have complex spatial heterogeneity under the effect of the external siphon effect (Liu and Xu 2021; Zhang et al. 2022; Yang et al. 2023; Chen et al. 2022; Deng et al. 2022) [15–19]. Therefore, it is necessary to study the spatial spillover of agricultural technological progress and the constraint mechanism of the agricultural operation scale.

The proposal of China’s rural revitalization strategy has attracted the attention of many Chinese scholars, who have tried to interpret this issue from the perspectives of agricultural technological progress efficiency, agricultural structure rationalization, agricultural carbon emissions, and even labor transfer (Hu 2018; Li and Zhou 2020; Ma and Kong 2019; Huang and Zhu 2022) [20–23]. However, by summarizing the existing research, it is not difficult to find that they have ignored the discussion of the threshold constraint role of the agricultural operation scale as a key variable, which is the starting point of the argument in this paper. In view of this, this paper intends to use the spatial panel data of 31 provinces in China (excluding Hong Kong, Macao and Taiwan) from 2007 to 2020 to test the nonlinear and spatial heterogeneity characteristics of agricultural technological progress on China’s interprovincial rural revitalization, further study the threshold mechanism of the agricultural operation scale, and explore its role in China’s rural revitalization to provide a theoretical reference and policy reference for further promoting the implementation of China’s rural revitalization strategy.

## Literature review and research hypotheses

The rural revitalization strategy being implemented in China essentially promotes the modernization of China’s rural agriculture. China’s rural society has undergone profound social changes, with the traditional rural acquaintance society tending to disintegrate, population mobility accelerating, and rural public nature gradually disappearing. How to keep the rural population alive and the economy vibrant and promote the endogenous development of the rural economy has become an important task in China’s modern rural construction. Therefore, it is necessary to use the promotion and application of agricultural technology as a tool and the rural economic community as the carrier to alleviate the structural embarrassment such as the lagging level of rural development and the weak endogenous development momentum. In this process, industrial prosperity is the focus, and agricultural technological progress is an important starting point, providing important strategic support for realizing the modernization of China’s agricultural industry and promoting rural revitalization (Zou 2018) [24].

From the traditional perspective, agricultural technological progress includes the invention, popularization, and application of agricultural technologies in the areas of machinery, cultivation, breeding, and biochemistry to improve the land, breeding technology, and pest control technology (Basso et al. 2016; Pinsonneault and Kraemer 1989) [25,26]. From a modern perspective, agricultural technological progress includes the development of technologies that promote the expansion, optimization, and value-added of the agricultural industry chain and ultimately realizes the sustainable development of the rural economy through agricultural technologies such as ecological, value-added, benefit-based, and high-tech agricultural management and agricultural resource allocation (Li and Guo 2022) [27].

Both classic economic theories and endogenous growth theories try to explain the relationship between technological progress and agricultural economic development and theoretically emphasize the role of technological progress in economic growth. However, it should be noted that due to the constraints of geographical location, economic location, resource endowment and even capital, labor and other agricultural production factors, there are significant differences between agricultural technological progress and economic development in different regions, which is mainly manifested in the spatial spillover, threshold characteristics and spatial heterogeneity of agricultural technological progress.

### Spatial spillover effects of technological progress in agriculture

Griliches (1990) argues that with the development of information technology and the facilitation of transportation conditions, advanced technologies in the agricultural production process are more likely to bring about technology demonstration and diffusion effects through learning and imitation among adjacent regions with similar soil, topographical, and climatic conditions [28]. Due to the open-air nature of agricultural production, it is easy for surrounding farmers to discover the status and benefits of advanced agricultural production technologies, and the social relationship of acquaintances in geographical proximity makes it more advantageous for farmers to adopt interactive forms such as on-the-spot experience learning and demonstrations (Li and Xu 2018) [29]. From the perspective of agricultural factor prices, whether it is labor-biased or capital-biased technological progress, the original combination of agricultural factors will change, and through the price transmission mechanism of the agricultural product market, the changes in agricultural production outside the region that are highly compatible with the structure of agricultural factors will be induced to have a trickle-down effect on the external social economy (Arrow 1962) [30], change the agricultural production in the surrounding areas, promote the extension of agricultural industry functions and industrial agglomeration, and revitalize agricultural ecological resources.

Based on this, Hypothesis H1 is proposed: there is a spatial spillover effect on the impact of agricultural technological progress on rural revitalization.

### The nonlinearity of the impact of agricultural technological progress on rural revitalization

Rural revitalization is essentially a process of agricultural modernization. The key to agricultural modernization lies in the application of modern agricultural technological progress to agricultural production and rural construction. The existing research results show that agricultural technological progress improves agricultural production efficiency and reduces agricultural production costs through the input of mechanical and biochemical elements, promotes the development of agricultural production, processing and circulation, promotes the extension of the agricultural industry chain and the expansion of industrial functions, and consolidates the foundation for the prosperity of rural industries. Agricultural technological progress has allowed rural residents to attain an affluent life by increasing agricultural output, improving the quality of agricultural products, enhancing the market competitiveness of agricultural products, and increasing farmers’ operating income and wage income (Dhehibi et al. 2020; Dong et al. 2021) [31–33].

However, there is a complex feedback mechanism between agricultural technology and rural revitalization. For example, classic dualistic economic theory and the new economic geography model have been used to explain the mechanism through which agricultural technological progress impacts rural labor transfer (Zhao and Zuo 2022) [34], and the substitution effect of agricultural technological progress has led to the release of more of the agricultural labor force with a zero marginal production contribution, resulting in the loss of human resources in rural areas, thus having a negative impact [35]. At the same time, scholars such as Misbahuzzaman (2016) and Achour et al.(2021) believe that due to the great differences in human capital, land resources, and economic conditions in different regions, the substitution of traditional agricultural technologies with agricultural technological progress will also bring unintended consequences to agricultural production [36,37]. Therefore, only when the accumulation of agricultural technology reaches a certain level can the agricultural technological progress exert a long-term positive impact by promoting the agglomeration of agricultural product production, processing and circulation and promote the extension of the agricultural industrial chain and the expansion of industrial functions.

In addition, Yang (2021) and Li and Wang (2023) considered the impact of agricultural progress factors on China’s rural ecological environment from the perspective of agricultural carbon emissions, and they believed that agricultural technological progress expanded the amount of planting and mechanization in the early stage of development and increased agricultural carbon emissions. Their arguments acknowledge to a certain extent the existence of a U-shaped relationship between agricultural technological progress and rural ecological environments [38,39]. Therefore, in the long run, agricultural technological progress can achieve efficient and low-consumption intensive growth of agriculture through the innovation of agricultural production science and technology, the promotion of agricultural information technology, and the improvement of agricultural experience management and avoid overdraft and overexploitation of agricultural resources, reduce environmental pollution, and promote the realization of ecological livability in rural society, and on this basis, help in the development of industries such as health care and ecotourism with natural scenery as the core in rural areas, and realize the sustainability of the rural social economy (Wang et al. 2017; Wang 2019; Li and Guo 2022; Geng and Zhang 2023) [40–43].

Based on this, Hypothesis H2 is proposed: there is a nonlinear relationship between agricultural technological progress and rural revitalization.

### Agricultural technological progress and the threshold constraints of *Land-Scale Operation*

The concept of agricultural-scale operation originates from economies of scale and mainly refers to business activities in which the optimal allocation of factors such as labor, capital and land in agricultural production is realized in the process of agricultural production by expanding the scale of agricultural production and operation to obtain scale benefits.

Due to the diversity of agricultural operators, the indicators of agricultural-scale operation are usually measured from the perspectives of agricultural input and agricultural output (Ju 2018; Zheng et al. 2023) [9,44]. Among them, land is the most basic and important input factor of agricultural production, and the increase in other agricultural production factors is mostly based on the premise of the expansion of the land operation area, so the index of agricultural input is measured according to land-scale operation (*SLO*). The index of agricultural output usually refers to the scale of sales of agricultural products, so this paper uses the level of agricultural-industry agglomeration *(AIG*) to measure it.

The academic community realizes that rural revitalization requires the help of agricultural technology, and the scale of agricultural operations is related to the application and promotion of new agricultural technologies in agricultural production (Zheng et al. 2023) [44]. Large-scale agricultural operation is not only related to overcoming many of the technical problems present in the process of agricultural production and operation but also restricts the promotion and application of agricultural technological progress in agricultural production and has become an important means for farmers to directly reduce agricultural production costs, promote agricultural production specialization, and promote high-quality agricultural development.

The development of modern agriculture must promote the large-scale operation of rural agriculture, and large-scale operations must meet certain external conditions, such as agricultural technological progress (Cloutier et al. 2019) [45]. In this regard, Wang et al. (2022) believe that there is a critical level in the relationship between the scale effect of *SLO* and agricultural technology and that the promotion and application of agricultural technology will not reduce the use of chemical fertilizers due to traditional experiences and habits of agricultural farming [46]. Only when the degree of land fragmentation is reduced, and the expansion of land reaches a critical point can *SLO* in China’s main grain-producing areas provide a realistic basis for the application of green agricultural technologies. Then, advanced agricultural technologies and management methods can be applied and promoted, thereby promoting the improvement of rural nonpoint source pollution (Xiang 2022) [47].

Therefore, Luo’s (2000) study found that the land yield rate does not change with the application of *SLO*, but the capital investment in large-scale agricultural machinery with physical inseparability has the most direct requirements for *SLO*, and only large-scale operations can reduce the unit cost of agricultural technology input, while an excessive land operation scale will face high supervision costs and reduce the potential benefits of specialized agricultural management technology [48]. Similarly, based on the “S” curve characteristics of agricultural technology diffusion, Liu found that the adoption of advanced agricultural technologies by large-scale operators is affected by the scale of land and believes that when *SLO* reaches an inflection point, the management cost of adopting agricultural technologies by large-scale operators will increase, while the income from the land will not expand accordingly, thus limiting the adoption of new technologies by agricultural operators (Liu et al. 2017) [49]. Ma et al. (2019) investigated the mechanism of "land transfer-scale management-agricultural environmental efficiency" and pointed out that *SLO* affects the innovation and adoption of green agricultural technology, while *SLO* without green technology will fall into the dilemma of rural environmental constraints, which further shows that there is a mutual constraint relationship between *SLO* and agricultural technological progress, and emphasizes the threshold role of *SLO* [50].

Based on this, Hypothesis H3 is proposed: the impact of agricultural technological progress on rural revitalization has a threshold effect based on the constraints of *SLO*.

### Agricultural technological progress and the threshold constraints of *Agricultural-industry Agglomeration*

*AIG* has a primary dependence on the natural resource endowment of the regional agglomeration to set agricultural planting, processing, and circulation links of the market integration organization network of the advanced process. The most obvious embodiment is the concentration of large-scale agricultural production organizations, agricultural leading enterprises, and agricultural service institutions in a certain region, which not only reduce the management cost of agricultural production and the policy cost of public agricultural technology promotion but also provide conditions for the popularization and promotion of new technologies.

With the improvement of the level of *AIG*, Asresu et al. (2022) found that the effect of large-scale operations can reduce the cost of adopting new agricultural technologies for farmers, provide stable business expectations and investment incentives for large-scale operators, and further improve the degree of *AIG* [51]. Taeko Hiroi, Zhang et al. argue that as an overall input, the application of new agricultural technologies in agricultural production means that it is difficult to transform them into other uses again, and the characteristics of this asset specificity require that the level of *AIG* be improved to improve the degree of organization, centralization, and specialization of agricultural production and to reduce the risks and costs of the application of new agricultural technologies in decentralized agricultural production [52,53]. Huang et al. (2022), Wang and Liu (2011), Miao et al. (2023) and other scholars found that industrial agglomeration leads to more demand for agricultural technology innovation, and *AIG* is conducive to strengthening the collaboration and cooperation of similar enterprises in different regions. In this regard, Liang argues from a sociological perspective that large-scale *AIG* tends to decommunize, which can easily lead to the marginalization and proletarianization of the small farmers in the village, resulting in unintended consequences for the rural society [12,54,55].

Based on this, this paper proposes Hypothesis H4: the impact of agricultural technological progress on rural revitalization has a threshold effect based on the constraints of *AIG*.

## Study design and model setting

### Variable measurement

#### Dependent variable

The dependent variable was the level of rural revitalization (*LRR*). The "20-character general policy" of the 19th National Congress of the Communist Party of China on the implementation strategy of rural revitalization covers five major indicators, and domestic scholars generally construct the rural revitalization index according to the five indicators, but the article believes that the rural revitalization index system constructed by predecessors has certain limitations, for example, many literatures use the number of administrative villages with planning to measure the rural governance level index, which is controversial. Therefore, with reference to the research results of Yin et al. (2024) and Bo et al. (2023) [56,57], this paper constructs an evaluation system for China’s rural revitalization with three connotations: industrial revitalization, ecological environment, and common prosperity, and uses the entropy weight method to determine the level of rural revitalization in the comprehensive index, as illustrated in Table 1.

**Table 1:** Evaluation index system and description of China’s rural revitalization level.

Subsystem	Dimension	Description of the Indicator
Industrial revitalization	Level of agricultural mechanization	Total power of agricultural machinery per capita (KW/person)
	Land productivity	The ratio of agricultural value added to total cultivated land (%)
	Agricultural labor productivity	Ratio of the added value of the primary industry to the rural population (%)
	Level of agricultural integration and specialization	Number of agricultural production service enterprises and agricultural processing service enterprises (pcs)
	Village-enterprise synergy capabilities	Number of village collective economic organizations and farmers’ professional cooperatives (pcs)
	Proportion of facility agriculture	The ratio of agricultural greenhouse production area to total cultivated land area (%)
Ecological environment	Green energy utilization	Solar water heater area per capita (square meters per 10,000 persons)
	Rural greening level	The ratio of green cover area to total rural area (%)
	Household waste disposal capacity	Ratio of the number of sanitation vehicles to the total rural population (%)
	Fertilizer use reduction	Intensity of chemical fertilizer used per unit cultivated area (tons per hectare)
	Pesticide use reduction	Intensity of pesticide used per unit cultivated area (tons per hectare)
Common prosperity	Relative poverty rate	Proportion of rural residents with subsistence allowances (%)
	Income level of rural residents	Per capita net income of rural residents (Yuan)
	Engel’s coefficient	Proportion of food consumption expenditure (%)

Industrial revitalization is at the core of rural revitalization. Therefore, drawing on the research of scholars such as Xu and Wang (2022), Zhang and Bai (2022), Yang et al. (2023), Luo et al. (2023),Lu and Zhao (2023), this paper selects three-level indicators from the perspectives of agricultural input and output capacity, agricultural integration, and village collective economic coordination ability. Protection of the ecological environment is a key task of rural revitalization [58–62]. Therefore, this paper refers to the research results of Huang (2018), Cui et al. (2020), Liu et al. (2023) and selects third-level indicators with the breakthrough point of agricultural green lifestyle and agricultural carbon reduction development [63–65]. Providing rural residents with Common prosperity is the fundamental purpose of rural revitalization, the income level, quality of life and relative income gap of rural residents are highlighted in the selection of the third-level indicators.

### Core explanatory variables

The core explanatory variable in this paper is the level of agricultural technological progress (*ATA*). Due to the fragmentation of agricultural production and the fragmentation of land, there is no significant economic efficiency of scale in the agricultural industry, so this paper uses the DEA-Malmquist index method, assuming that the return to scale is constant, to estimate the degree of substitution of labor by agricultural technological progress (Anselin 2001; Tientao et al. 2016; Kuang et al. 2022) [66–68], that is, agricultural total factor productivity (*TFP*). At the same time, since *TFP* is a dynamic month-on-month index, it needs to be converted into a static fixed-base index to reflect the level of agricultural total factor productivity. Therefore, this paper sets 2006 as the base period, and the base period value of agricultural total factor productivity in each province is 1. Then, it uses the index multiplication method to calculate the level of agricultural total factor productivity in each period and uses it as a proxy variable to measure the level of agricultural technological progress.

The expression is as follows:

$$\begin{array}{l} TFPCH=M_{i}^{t}(y_{t},x_{t},y_{t+1},x_{t+1})\\ \;={\left[\frac{D_{i}^{t}(y_{t+1},x_{t+1})}{D_{i}^{t}(y_{t},x_{t})}\times \frac{D_{i}^{t+1}(y_{t+1},x_{t+1})}{D_{i}^{t+1}(y_{t},x_{t})}\right]}^{\frac{1}{2}}\\ \;=\frac{D_{i}^{t+1}(y_{t+1},x_{t+1})}{D_{i}^{t}(y_{t},x_{t})}\times {\left[\frac{D_{i}^{t}(y_{t+1},x_{t+1})}{D_{i}^{t+1}(y_{t+1},x_{t+1})}\times \frac{D_{i}^{t}(y_{t},x_{t})}{D_{i}^{t+1}(y_{t},x_{t})}\right]}^{\frac{1}{2}} \end{array}$$ (1)

Eq (1) includes the rate of agricultural technological progress and the efficiency of agricultural progress in a narrow sense, where *M* is the rate of change of total factor productivity, and *x* and *y* are the inputs and outputs in the period *t* and *t+1*, respectively. In this study, the gross output value of agriculture, forestry, fishery and animal husbandry was set as the output variable of the DEA-Malmquist model, the price factor of the gross output value in the later stage was deflated (constant price in 2007), and the rural electricity consumption, the total area of rural cultivated land, the total power of agricultural machinery and the number of rural employees were taken as the input variables of the DEA-Malmquist model.

### Threshold variables

The threshold variables used in this study are *SLO* and *AIG*.

Land-scale operation is indeed a problem of land area in the literal sense but considering the current situation of land fragmentation in China, it is somewhat inappropriate to measure land scale management based on per capita land area. and Considering that the expected investment risk and operating income of Land-scale operation will touch on the issue of rural land property rights and that the confirmation of land contract management rights certificates will stabilize the land contract relationship, enhance the sense of security of business entities in scale operation, and encourage business entities to make long-term land investments, this paper adopt the research methods of predecessors and uses the number between 2007 to 2020 of rural land contract management rights certificates issued by local governments to measure *SLO* (Yang and Zhang 2023; Shi and Zhang 2022) [69–72], *AIG* refers to the research method of Miao et al. (2023). Yang and Zhang (2023) in the research process and uses location entropy to calculate the regional concentration of the agricultural industry [55,69].

The specific calculation method is shown in Eq (2) as follows:

$$AIG_{it}=\frac{A_{it}/G_{it}}{A_{t}/G_{t}}$$ (2)

Among them, *AIG* represents the level of *AIG*, *A* represents the gross output value of agriculture, forestry, fishery and animal husbandry, *G* is the *GDP*, *i* represents the region, and *t* is the year.

### Control variables

To avoid the problem of endogeneity caused by the omission of relevant explanatory variables, this study drew on the existing research results of Hu (2018), Ma and Kong (2019), Zhang and Bai (2022). and introduced factors that may affect rural revitalization as the control variables for the regression analysis of the model in this study [20,22,59].

1. Rural human capital *(FKL)*. This paper uses the average number of years of education in rural areas to represent rural human capital, that is, the ratio of those with a junior high school education or above to the population aged 6 and above.
2. The level of financial support for agriculture (*FIN*). This paper uses the ratio of the balance of agriculture-related loans of financial institutions to the gross output value of agriculture, forestry, fishery and animal husbandry in each region to measure the level of financial support for agriculture.
3. The level of fiscal support for agriculture *(AIP*). The scale of expenditure on agriculture, forestry and water affairs reflects the degree of support for agriculture in various provinces, so the ratio of expenditure on agriculture, forestry and water affairs to the gross output value of agriculture, forestry, fishery and animal husbandry is used to express the level of fiscal support for agriculture.
4. The degree of disaster on Agriculture (*DDA*). The development of the rural economy is largely affected by natural disasters. The frequent occurrence of natural disasters not only destroys rural infrastructure and increases the difficulty of agricultural production, but also seriously impacts the livelihood sources of rural households, increases the uncertainty of agricultural inputs, inhibits farmers’ expectations for production in the long run, and strengthens the trend of rural labor outflow. Therefore, the degree of disaster on Agriculture is included in the control variables, and the ratio of the affected area to the cultivated land area in each region is used to measure it.

### Research methods and models

#### Kernel density estimation method

In this study, the kernel density estimation method was used to investigate the spatial heterogeneity of China’s rural revitalization level and agricultural technological progress. Kernel density estimation assumes that the variable to be estimated obeys a certain probability density function distribution, abandons the various assumptions of the parameter estimation method, and uses a continuous curve to describe the dynamic trend of the variable to be estimated over time, which has unparalleled advantages in analyzing large sample data. The common Gaussian kernel function (as shown in Eq (3)) is used to estimate the density value, and the adaptive kernel density function of Eq (4) is used to estimate the probability of the variable being estimated at a certain value.

$$K(x)=\frac{1}{\sqrt{2\pi }}\exp(-\frac{x^{2}}{2})$$ (3)

$$f(x)=\frac{1}{N\rho }\sum_{i=1}^{n}K(\frac{x_{i}-\bar{x}}{\rho })$$ (4)

where *K(·)* is the Gaussian kernel function, *x*_*i*_ is the variable to be estimated, *f(x*_*i*_*)* is the kernel density function, *ρ* is the optimal bandwidth, and *N* is the number of samples observed.

#### Exploratory Spatial Data Analysis (ESDA)

Moran’s I is generally needed to identify the spatial correlation of variables before using a spatial econometric model to test spillover effects. The formula for calculating Moran’s I is as follows in Eq (5):

$$Moran^{\prime }sI=\frac{\sum_{i=1}^{n}\sum_{j=1}^{n}W_{ij}(Y_{i}-\bar{Y})(Y_{j}-\bar{Y})}{\frac{\sum_{i=1}^{n}{\left(Y_{i}-\bar{Y}\right)}^{2}}{n}\sum_{i=1}^{n}\sum_{j=1}^{n}W_{ij}}$$ (5)

where *W* is the weight and *Y* is the test variable.

In this study, the robustness of the model is tested by replacing the spatial weight matrix, so two spatial weight matrices are used in the test process: the first is the adjacency weight matrix *W1* with the adjacency of two places as the value criterion (0–1), and the second is the economic and geographical weight matrix *W2* with per capita *GDP* as the weight.

$$W_{2}=W_{1}\times E\;E_{ij}=\{\begin{array}{c} \frac{1}{\left|{\bar{Y}}_{i}-{\bar{Y}}_{j}\right|},i\neq j\\ 0,i=j \end{array}$$

where *Yi* is the per capita *GDP* of the sample spatial unit and $\bar{Y}$ is the mean.

#### Spatial Durbin Model (SDM)

Spatial econometric models mainly include the spatial Durbin model (SDM), spatial lag model (SAR) and spatial error model (SEM). SAR is commonly used in the academic community to analyze whether there is a spatial spillover effect on variables, and SEM is used to analyze the heterogeneity of spatial interactions caused by location differences. The specific formula is as follows:

$$SAR:Y_{i,t}=\alpha_{0}+\rho WY_{i,t}+\sum_{j=1}^{n}\alpha_{j}X_{j,t}+\varepsilon_{i,t}$$ (6)

where *i* and *t* represent the observed individual and the observed year, *Y* is the explanatory variable, *X*_*j*,*t*_ is the explanatory variable, *ɑ*_*0*_ is the intercept, *ε*_*i*,*t*_ is the random error term, *ɑ*_*i*_ is the regression coefficient, *ρ* is the spatial autoregressive coefficient, and *W* is the spatial weight matrix.

$$SEM:Y_{i,t}=\alpha_{0}+\sum_{j=1}^{n}\alpha_{j}X_{j,t}+\lambda W\mu_{it}+\varepsilon_{it}$$ (7)

where *λ* is the spatial error coefficient and the perturbation term *u*_*it*_ is assumed to have a spatial correlation.

$$SDM:Y_{i,t}=\alpha_{0}+\sum_{i=1}^{n}X_{i,t}+\rho WY_{i,t}+\theta W\sum_{i=1}^{n}X_{i,t}+\varepsilon_{i,t}$$ (8)

where *θ* and *ρ* are the regression coefficients of the spatial lag terms of the explanatory variable and the explanatory variable, respectively.

In the empirical process, the results of the LM test, LR test and Wald test of the model should be used to determine whether the SDM model will degenerate into SAR or SEM, and then the selection of random effect and fixed effect models should be judged according to the results of Hausman’s test.

To verify the research hypothesis proposed in this paper, this study selected China’s interprovincial panel data from 2007 to 2020 as the research sample and constructed a spatial panel model to analyze the impact of agricultural technological progress on China’s interprovincial rural revitalization. At the same time, considering that the impact of agricultural technological progress on China’s interprovincial rural revitalization is not linear, the square term of the agricultural technological progress index is added to the estimation process, and the SDM model is set as shown in Eq (9).

$$\begin{array}{l} SDM:LRR_{i,t}=\alpha +\rho \sum_{j=1}^{n}\omega_{i,t}LRR_{i,t}+\beta (ATA_{i,t}+AT{A_{i,t}}^{2}+\sum_{i=1}^{n}D_{i,t})\\ \;+\theta \sum_{j=1}^{n}\omega_{i,t}(ATA_{i,t}+AT{A_{i,t}}^{2}+\sum_{i=1}^{n}D_{i,t})+c_{i}+\mu_{t}+\varepsilon_{i,t} \end{array}$$ (9)

where *D* represents the control variable, *β* is the coefficient to be estimated, *ρ* is the coefficient to be estimated for spatial autocorrelation, *c*_*i*_ and *μ*_*t*_ represent the province and time fixed effects, respectively, and *ε*_*i*,*t*_ is the error term. *ω*_*it*_ is the spatial weight matrix, and *θ* is the coefficient vector of the spatial spillover effect.

#### Threshold effect model

The threshold effect model is set up based on Hansen’s (1999) method to explore the piecewise function relationship between agricultural technological progress and interprovincial rural revitalization in China by finding the threshold value of the agricultural operation scale, and the panel threshold model is set as follows:

$$LRR_{it}=\mu_{i}+\beta_{1}^{\prime }ATA_{it}\cdot I(q_{it}\leq \gamma )+\beta_{1}^{\prime \prime }ATA_{it}\cdot I(q_{it}>\gamma )+\sum \kappa D_{it}+\varepsilon_{it}$$ (10)

where *D* is the model control variable, I(·) is the representative function, is the threshold value, which is the threshold variable *SLO* and *AIG*. is the coefficient to be evaluated for the threshold model.

where *D* is the model control variable, *I(·)* is the indicative function, *γ* is the threshold value, and *q* is the index of the threshold variable, *SLO* and *AIG*. $\beta_{1}^{\prime },\beta_{1}^{\prime \prime },\kappa$ is the coefficient to be evaluated for the threshold model.

## Data sources and characteristic facts

### Description of the data source

Due to the availability of data, the scope of this study covers 31 provincial-level administrative regions in the Chinese mainland but does not include Hong Kong, Macau and Taiwan. Among them, the data of the rural revitalization evaluation system are from the "China Rural Management Statistical Yearbook", "China Rural Statistical Yearbook", "China Urban and Rural Construction Statistical Yearbook", "Chinese Population and Employment Statistical Yearbook" and the national greenhouse data system. The descriptive statistics of the data are shown in Table 2.

**Table 2:** Descriptive statistics of the variables.

Type of Indicator	Symbol	Sample Size	Mean	Standard Deviation	Min.	Max.
Explained variable	*LRR*	434	0.312	0.109	0.130	0.753
Explanatory variables	*ATA*	434	1.443	0.259	0.046	2.935
	*FKL*	434	5.311	1.211	1.209	8.789
	*FIN*	434	2.467	2.159	0.049	13.411
	*AIP*	434	0.254	0.331	0.034	2.091
	*DDA*	434	0.303	0.253	0.008	1.598
Threshold variables	*SLO*	434	0.094	0.065	0.004	0.600
	*AIG*	434	1.189	0.514	0.038	3.295

Source of data: Actual measurements.

To verify the rationality of the setting of China’s rural revitalization index system, this paper uses the questionnaire survey method to set up evaluation questions for the rural revitalization index system and compares the weights with the first-level indicators of rural revitalization through the TOPSIS entropy weight method. The results are within the acceptable range, so the data used in the article are still dominated by yearbook data.

**Fig 1:**
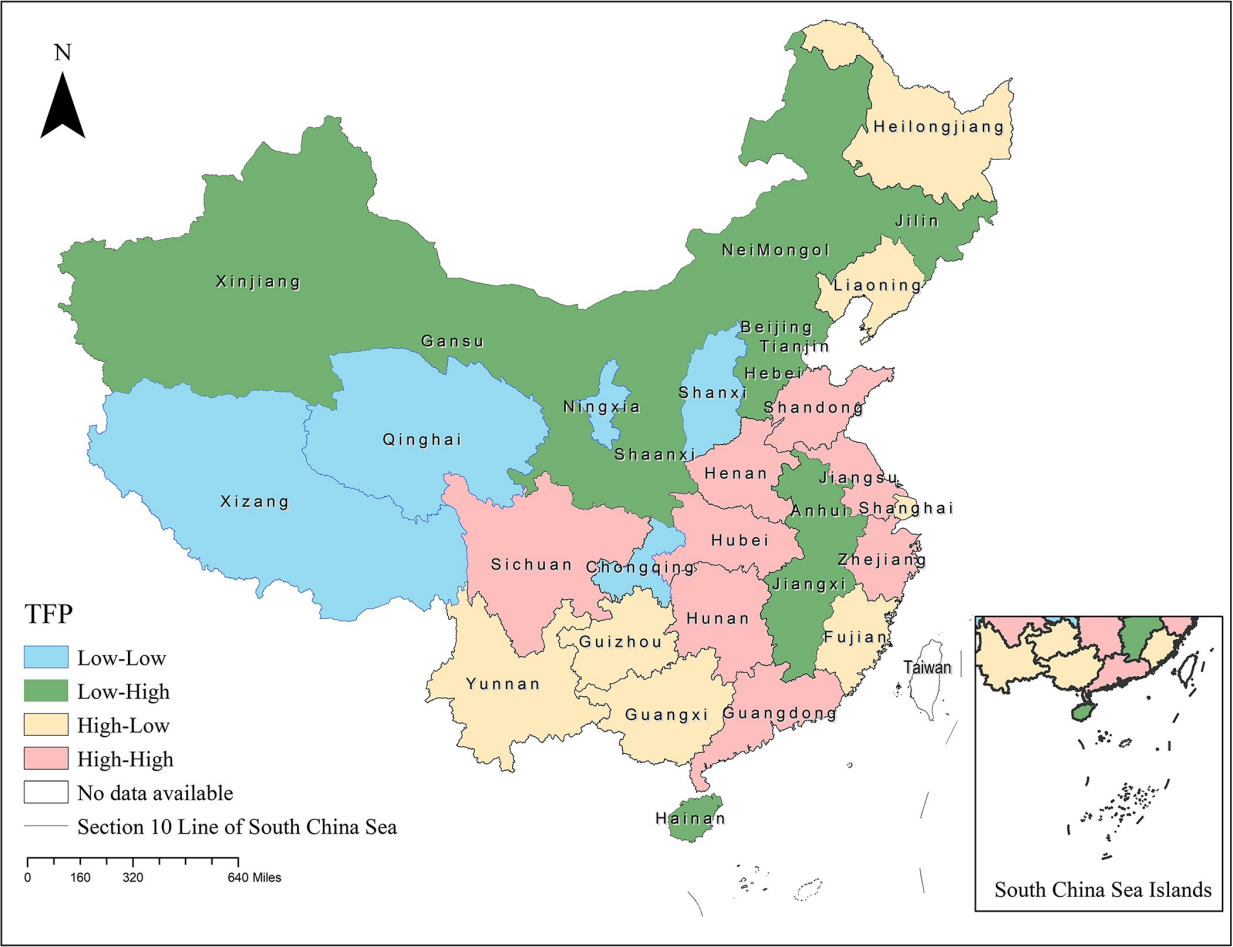
Spatial distribution of cold and hot spot agglomeration areas on the mean of interprovincial agricultural technological progress in China, 2007–2020. Note: This map was created using ArcGIS 10.2 software, and the base map was taken from the Natural Earth (http://www.naturalearthdata.com).

*Preliminary law description of interprovincial agricultural technological progress in China*. In this paper, ArcGIS 10.2 spatial geographic statistical analysis software is used to visualize the TFP change rate of interprovincial agricultural total factor productivity in China from 2007 to 2020, as shown in Fig 1 below.

As seen from Fig 1, China’s agricultural technological progress as a whole show’s significant spatial heterogeneity, and the hot spots and subhot areas are mostly concentrated in the central and eastern parts of China.

First, the central and eastern regions of China, such as Shandong, Jiangsu, and Zhejiang, are mostly hot spots and subhot spots. These provinces are both traditional agricultural provinces and provinces with outstanding industrial and commercial development. The above areas focus on the deep processing of agricultural products, deepen the agricultural industry chain, promote the development of agricultural industrialization, use modern equipment in agricultural production, promote agricultural mechanization, and promote the rapid progress of agricultural technology development. The competitiveness of the agricultural industry continues to be highlighted, unlike in Jiangxi and Fujian, which are mostly hilly and mountainous areas. As a major agricultural province, Anhui is in an insignificant area, reflecting a lack of agricultural input, and the impact of extreme floods cannot be ignored.

Second, Shanxi, Ningxia, Gansu and other western regions are in cold spot and subcold spot areas on the spatial agglomeration map of agricultural total factor productivity, and from the statistical data, the provinces in the abovementioned agricultural ecologically fragile areas are susceptible to the impact of farmland water conservancy and natural disasters, various agricultural input factors are not fully utilized or effectively allocated, and the change rate of agricultural factor productivity is at a low level, which also reflects that the agricultural technology promotion and application system at the rural grassroots level still needs to be further improved.

#### Characterization of the heterogeneity of interprovincial rural revitalization in China

**Fig 2:**
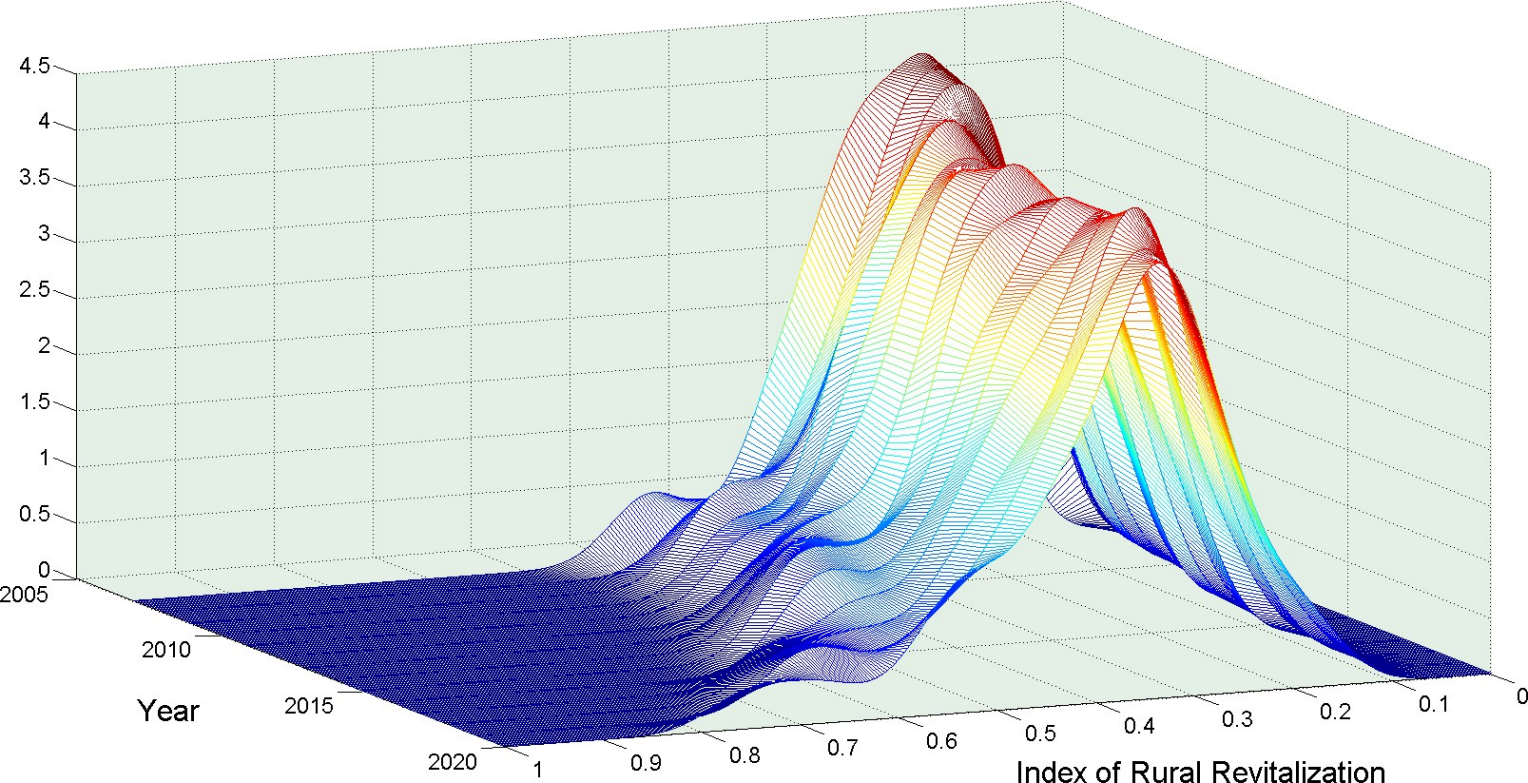
Three-dimensional plot of the kernel density estimation of the interprovincial rural revitalization level in China from 2007 to 2020.

With the help of MATLAB tools, this paper analyzes the dynamic changes in China’s rural revitalization level by using the kernel density estimation method, and the results are shown in Fig 2.

The figure displays the following information regarding the spatiotemporal and spatial distribution dynamics of the rural revitalization levels in 31 provincial-level administrative regions in China from 2007 to 2020.

It can be seen above figure first that the peak height of the kernel density function gradually decreased, evolving from steep to gradually flattened, and the left trail first shifted to the left and then gradually shifted to the right, which means that the level of interprovincial rural revitalization in China has a differentiated trend. Second, the position of the main peak of the kernel density function continues to shift to the left, but the displacement is not obvious, so it can be judged that although the overall level of interprovincial rural revitalization in China has been continuously improved, the improvement is not obvious. Finally, although the number of main peaks in the kernel density function does not change, the number of peaks gradually increases, which means that although the levels of rural revitalization of provinces in China shows a differentiated trend, the polarization trend is not obvious, indicating that although there is a gap between regions in China’s rural revitalization, the gap does not change in the direction of deterioration.

The spatial distribution pattern of inter-provincial rural revitalization in China means that the trend from convergence to dispersion is decreasing, especially in recent years, the map of kernel density distribution has gradually flattened, and the bimodal has become increasingly obvious. This is due to the lag in rural development and imperfect infrastructure in most provinces of China, which has a large gap with the eastern coastal areas, but it can be seen from the kernel density distribution map that it is gradually moving to the left, which also shows that the central and western regions of China are catching up strongly with the eastern region, especially when the development of the three northeastern provinces of China is slowing down. However, the intensification of population migration, the expansion of fiscal and capital investment in the central and eastern regions, and the frequent occurrence of extreme meteorological disasters caused by climate change have widened the disparities between rural areas in China in recent years. In particular, with the acceleration of the trend of land circulation in China, some provinces have intensively used land to develop economies of scale, strengthened the construction of rural infrastructure and public services, used new agricultural technologies to develop new agriculture, smart agriculture and characteristic agriculture, and extended the agricultural industry chain to improve agricultural production income, which has also contributed to the increasingly significant differences between rural regions in China.

## Results of econometric analysis

### Test results of the spatial effect of rural revitalization

Spatial econometric analysis first tests whether there is a spatial correlation, and in general, Moran’s I index is usually used to explain the spatial correlation problem.

**Fig 3:**
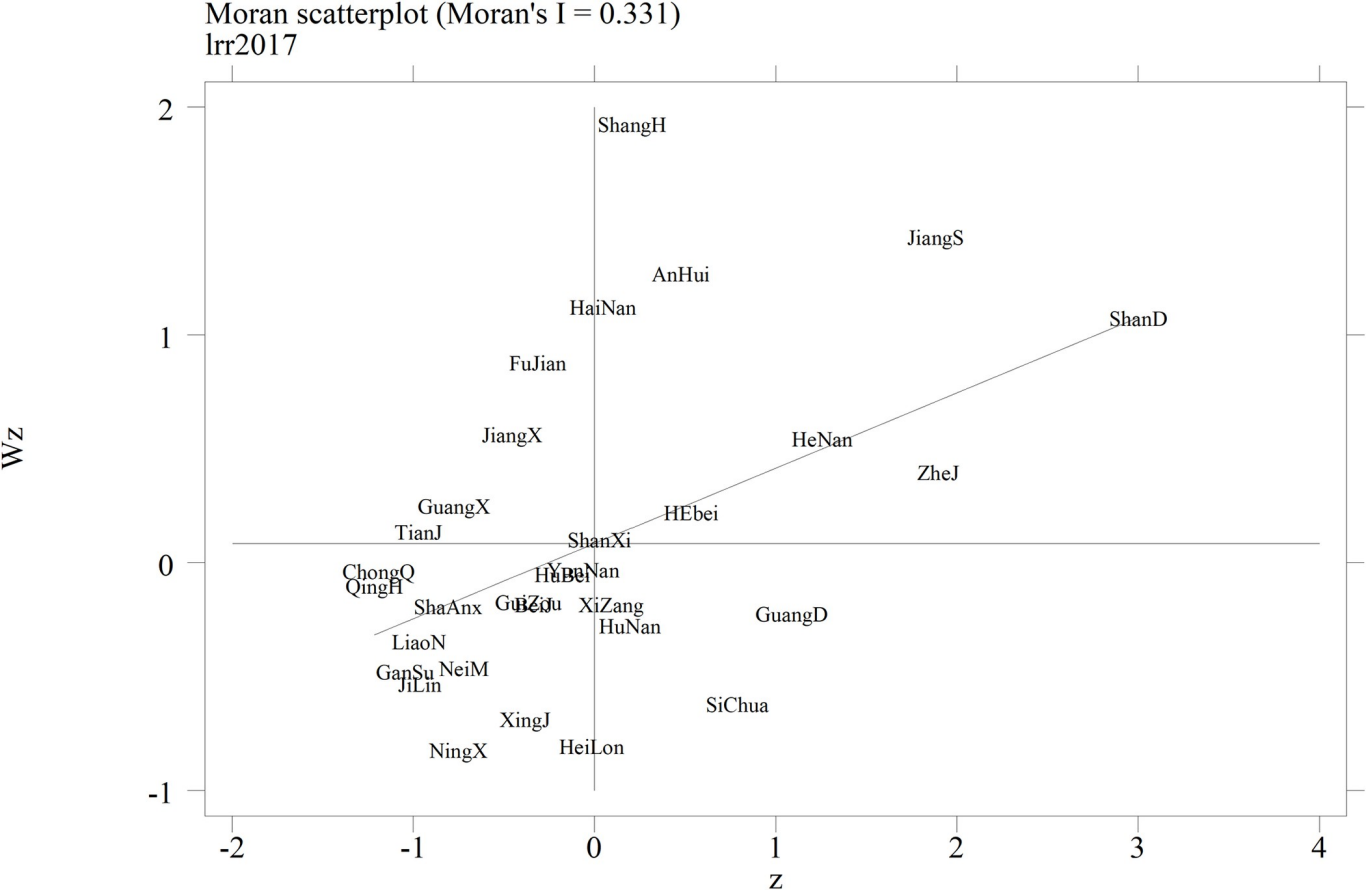
Scatterplot of the local spatial correlation of rural revitalization in China.

This paper finds that the global autocorrelation Moran’s I index of China’s interprovincial rural revitalization level fluctuates between 0.253 and 0.378 from 2007 to 2020, showing a gradual downward trend overall. The I index gradually increased and passed the significance test (Fig 3), which means that there is a significant spatial correlation and obvious spatial spillover effect at the level of interprovincial rural revitalization in China, but the phenomenon of geographical agglomeration is gradually weakened, the interregional connection is gradually enhanced, and the problem of regional development imbalance is improved.

### The spatial panel model test

Therefore, it is reasonable to choose a spatial econometric model to carry out research on agricultural technological progress and rural revitalization.

According to the results of the spatial correlation test, there is a significant positive spatial correlation between the levels of rural revitalization in China, and to alleviate the estimation bias caused by model selection, it is necessary to construct a spatial econometric model to estimate the spatial spillover effect of agricultural technological progress on rural revitalization.

As seen from Table 3, first, the Hausman test rejects the null hypothesis at the significance level of 1%, and the fixed-effect model should be selected for Model (2), Model (3), and Model (4).

**Table 3:** Spatial dubin model test and regression results.

Variables and statistical parameters	Model testing		SDM model regression results	
	Mixed-effects model (1)	SDM (2)	Static SDM (3)	Dynamic SDM (4)
*L*.*LRR*				0.4366***
				(0.0523)
*ATA*	0.0141	-0.0077*	-0.0435*	-0.0728**
	(0.0103)	(0.0041)	(0.0156)	(0.0302)
*ATA* ^*2*^	0.0307**	0.02655**	0.0429**	0.0841**
	(0.0154)	(0.0125)	(0.0177)	(0.0398)
*ATA×SLO*	0.0133***			
	(0.0034)			
*ATA×AIG*	0.0076***			
	(0.0014)			
Control variables	NO	NO	YES	YES
Time fixation effect		YES	YES	YES
Regional fixed effects		YES	YES	YES
*Spatial rho*		0.5148**	0.3892***	0.4217***
		(0.2308)	(0.0576)	(0.0416)
*W×ATA*		-0.0270	-0.0308**	-0.0619***
		(0.0167)	(0.0138)	(0.0115)
*W×ATA* ^*2*^		0.0225**	0.1069***	0.0571**
		(0.0097)	(0.0086)	(0.0235)
*LM-lag*		62.67***		
*Robust LM-lag*		0.4396		
*LM-error*		45.59*		
*Robust LM-error*		11.03**		
*SLM*:*LR test*		31.68***		
*SLM*:*Wald test*		28.66***		
*SEM*:*LR test*		46.37***		
*SEM*:*Wald test*		55.21**		
*Husman Test*	12.39**	8.77**	9.41**	14.35**
*Sigma2_e*		0.029***	0.048***	0.066***
*R2*	0.364	0.415	0.497	0.554
Obs	434	434	434	434

Note

***, **, and * represent statistical significance at the 1%, 5%, and 10% levels, respectively. Standard errors are in parentheses. The following table notes are the same.

Second, according to the results of the LM and robust-LM tests, the LM(error) and R LM(error) of the spatial error SEM passed the significance level test of 1%, the LM(lag) of the spatial lag model (SLM) passed the significance level test of 1%, the robust-LM(lag) did not pass the significance test, and the Moran’s I index was 7.739, which was significant at the 1% test level. Therefore, it is preliminarily judged that the spatial effect of the impact of agricultural technological progress on rural revitalization cannot be ignored, and the spatial Durbin model should be used for model selection.

In addition, the results of Wald’s test and LR test further verify the above judgment, and Model (2) rejects the null hypothesis at the 1% significance level, which means that the spatial Durbin model cannot be degenerated into the SLM and SEM.

### Analysis of the empirical results of the spatial durbin model

The results in Table 2 show that the ATA coefficient of the mixed-effect Model (1) is estimated to be positive and fails to pass the significance test, which shows that the model estimate that ignores the spatial spillover effect of H1 is biased, and the coefficient estimation of the ATA square term and the interaction term is significantly positive, which means that the impact of agricultural technological progress on regional rural revitalization presents a weak "U" shaped relationship. This shows that under the influence of low-level agricultural technology, China’s rural revitalization will face the problem of labor substitution, and only when the accumulation of agricultural technology reaches a certain level will rural revitalization be significantly improved with the adjustment of other factors in the rural economy, such as *SLO* and *AIG*.

Furthermore, Columns (2), (3) and (4) in Table 2 show that under the influence of control variables and spatial effects, the goodness-of-fit of the spatial Durbin model has been significantly improved, and the degree and direction of the impact of agricultural technological progress on rural revitalization have become increasingly clear. The results of the fixed-effect spatial Durbin Model (2), the static Model (3) and the dynamic spatial Durbin Model (4) all show that the direct effect coefficient of agricultural technological progress is significantly positive, which reflects the direct impact of agricultural technology imitation between adjacent regions on rural revitalization under spatial autoregression.

The explanation is that the progress of agricultural technology has produced labor substitution, which complicates the development of the local rural economy in the context of labor transfer but eliminates the adverse effects of labor loss through *SLO* and industrial factor agglomeration to play a positive role in local rural revitalization. The spatial hysteresis coefficient is negative in the static space Durbin model and the dynamic space Durbin model test, and the spatial spillover coefficient represents the spatial spillover of technology diffusion under the influence of the trickle-down effect and echo effect, and its value is negative; that is, the improvement of the local technology level will produce an economic siphon on the production factors of the neighboring areas, lead to the loss of labor, capital and other production factors in the neighboring areas and even the peripheral areas, weaken the driving force of agricultural industrialization, and reduce the enthusiasm of the financial agricultural input of the neighboring areas, so it shows an inhibitory effect.

Based on the above analysis, it is assumed that H1 and H2 is confirmed.

Strictly speaking, however, there is a slight difference between the estimation coefficients of the spatial lag variable and the spatial spillover effect. Pace and LeSage (2007) [73] suggested that direct and indirect effects should be used to measure the spatial spillover effects of explanatory variables, and this paper uses the partial differential equation decomposition method to decompose the spatial spillover effects of explanatory variables into direct and indirect effects. As seen in Table 4 below.

**Table 4:** Decomposition results of spatial effects of SDM.

Variable	Direct effects		Indirect effects		Total effect	
	W_1_	W_2_	W_1_	W_2_	W_1_	W_2_
*ATA*	-0.0761**	-0.0701***	-0.0304	-0.0476	-0.1068*	-0.1171**
	(0.0336)	(0.0255)	(0.0214)	(0.0036)	(0.0574)	(0.0552)
*ATA* ^*2*^	0.0134**	0.0248*	0.0260**	0.0255**	0.0387***	0.0495**
	(0.0065)	(0.0133)	(0.0115)	(0.0115)	(0.0116)	(0.0213)
*FKL*	0.0637*	0.1502*	0.2245	0.5148	0.2872*	0.6667***
	(0.0359)	(0.0853)	(0.1421)	(0.3237)	(0.1631)	(0.1554)
*FIN*	0.0166	0.0306**	0.0056	0.0170	0.0201	0.0485*
	(0.0134)	(0.0685)	(0.0045)	(0.0165)	(0.0177)	(0.0265)
*AIP*	0.0581**	0.1127***	-0.0241	-0.0436	0.0345	-0.0693*
	(0.0238)	(0.0239)	(0.0317)	(0.0307)	(0.0214)	(0.0395)
*DR*	-0.0082**	-0.0011**	-0.0209**	-0.0144*	-0.0286*	-0.0155**
	(0.0035)	(0.0003)	(0.0106)	(0.0077)	(0.0152)	(0.0073)

Note: W_1_ represents the 0–1 adjacency matrix; W_2_ represents the geographic distance matrix.

Table 4 shows that the primary term of agricultural technological progress is negative in the direct effect, indirect effect and total effect under the two spatial weight matrices and passes the 10% significance test except for the indirect effect, which is not significant.

The direct effect of agricultural technological progress on rural revitalization is negative, which means that the negative impact of the transfer of production factors due to labor substitution on rural revitalization cannot be ignored, the square term of agricultural technological progress is significantly positive under the two spatial weight matrices, and the indirect effect is greater than the direct effect, indicating that the demonstration diffusion effect of new technologies can play a stronger radiation driving role, and the technology introduction, technology diffusion and even innovation activities generated by the forward and backward correlation of agricultural industry between regions can eliminate the restraints of administrative boundaries. Dismantling the boundaries of the market will help improve the rural revitalization levels in neighboring areas.

In terms of control variables, the indirect effect of human capital is not significant, the spillover to neighboring land caused by the relationship of talent competition between regions is not obvious, the indirect effect of financial support for agriculture due to financial siphoning is not significant, the agricultural industry policy variable will cause local residents to increase their tendency to transfer to the outside world to a certain extent due to the improvement of the public infrastructure and public services in "neighboring" villages, and the indirect effect is not significant.

### Robustness test

In the robustness test, this paper selects the economic geospatial matrix to replace the adjacent spatial weight matrix used in the previous paper, deletes the municipality sample, adjusts the sample time span to 2010–2020, and then estimates again, and the estimation results are shown in Table 5, Columns (5), (6), and (7).

**Table 5:** Dynamic space dubin model robustness test regression results.

Variables and statistical parameters	Shorten the sample time span (5)	Replace the sample (6)	Replace the spatial matrix (7)
*L*.*LRR*	0.1902*	0.1147*	0.2062***
	(0.1033)	(0.0635)	(0.0414)
*ATA*	-0.0076*	-0.0864	-0.0013**
	(0.0040)	(0.0787)	(0.0010)
*ATA* ^*2*^	0.0153*	0.0186**	0.0243***
	(0.0086)	(0.0414)	(0.0042)
Control variables	YES	YES	YES
Time fixation effect	YES	YES	YES
Regional fixed effects	YES	YES	YES
*Spatial rho*	0.1022**	0.0391**	0.0577***
	(0.0454)	(0.0179)	(0.0075)
*W×ATA*	-0.0064*	-0.0122	-0.0062*
	(0.0031)	(0.0136)	(0.0032)
*W×ATA* ^*2*^	0.0205***	0.0645**	0.0724**
	(0.0054)	(0.0274)	(0.0274)
*Sigma2_e*	0.0041***	0.0603***	0.0052***
	(0.0011)	(0.0092)	(0.0011)
*R2*	0.485	0.249	0.351
Obs	310	434	434

In Table 5, Column (5), the time span of the sample is adjusted to 2010–2020, and the estimation results show that the estimation coefficients of the primary and secondary terms of agricultural technological progress are significant within the 10% level, which is basically in line with the previous estimation results. In Table 5, Column (6), the sample of municipalities directly under the central government were deleted, and the estimation results showed that the estimation coefficient of the secondary item of production agricultural technology was significant within the 5% level. Furthermore, Table 5, Column (7) shows that the impact of agricultural technological progress on rural revitalization is weakened, which means that the large economic gap in adjacent areas will limit the absorption capacity of technology spillover, thereby inhibiting the knowledge transfer and diffusion of agricultural technology into neighboring areas, but the estimation results show that the direction and significance of the effect are basically the same, which means that the above estimation results and research conclusions are relatively stable.

### Heterogeneity test

To investigate the spatial heterogeneity of the impact of agricultural technological progress on China’s interprovincial rural revitalization, this paper first sorted the 31 administrative regions into major agricultural provinces and nonagricultural provinces according to the average added value of agriculture, forestry, animal husbandry and fishery from 2007 to 2020 and then divided the sample areas into eastern, central and western regions according to geographical location. The results of the dynamic spatial Durbin model test are shown in Table 6, Columns (8) to (12) below.

**Table 6:** Results of agricultural technological progress on rural revitalization by region.

Variables and statistical parameters	Major grain producing areas(8)	Nonmajor grain producing areas (9)	Western (10)	Central (11)	Eastern (12)
*L*.*LRR*	0.412***	0.205***	0.140***	0.226***	0.596***
	(0.029)	(0.049)	(0.015)	(0.036)	(0.104)
*ATA*	-0.032***	-0.068	-0.013	-0.029**	0.017***
	(0.005)	(0.064)	(0.012)	(0.015)	(0.002)
*ATA* ^*2*^	0.047***	0.023**	0.012**	0.042**	0.008
	(0.005)	(0.011)	(0.006)	(0.018)	(0.013)
Control variables	YES	YES	YES	YES	YES
Time fixation effect	YES	YES	YES	YES	YES
Regional fixed effects	YES	YES	YES	YES	YES
*Spatial rho*	0.075**	0.078**	0.092**	0.063***	0.104***
	(0.036)	(0.038)	(0.041)	(0.005)	(0.025)
*W×L*.*LRR*	0.004**	0.075**	-0.101	0.071*	0.067**
	(0.002)	(0.032)	(0.091)	(0.041)	(0.029)
*W×ATA*	-0.011***	-0.042*	-0.025	-0.007	0.0251**
	(0.004)	(0.023)	(0.020)	(0.015)	(0.010)
*W×ATA* ^*2*^	0.072**	0.026**	0.022**	0.081***	0.060**
	(0.011)	(0.013)	(0.011)	(0.017)	(0.026)
*R2*	0.5715	0.2644	0.4640	0.5214	0.6769
Obs	210	224	140	140	154

Table 6 shows that the lag terms of interprovincial rural revitalization in China are significantly positive, and the spatial spillover terms *(W×L*.*LRR)* pass the significance test at the level of 5%, except for the western region, which indicates that there is a spatial spillover effect on interprovincial rural revitalization in China. For large agricultural provinces, the curve of the impact of agricultural technological progress on the level of rural revitalization is "U" shaped. This indicates that there is spatial heterogeneity in the promotion of agriculture by traditional agricultural technologies such as agricultural mechanization, pest control and seed improvement, while the roles of precision agriculture, packaging agriculture, tourism agriculture and modern agricultural technologies with specialization and brand management are consistent with the direction of China’s interprovincial rural revitalization.

The direct effect of agricultural technological progress on the level of rural revitalization in the central and western regions showed a “U” shaped trend, and the spatial effect *W×ATA* was negative, which means that the labor substitution effect of agricultural technological progress has led to the agglomeration of agricultural production factors in eastern China, and the progress of agricultural technology in the eastern region has an economic siphon effect on the central and western regions. The spatial effect of *W×ATA*^*2*^ in the three regions is significantly positive, which means that Land-scale operation, agricultural industry agglomeration, the cross-regional operation of large-scale advanced agricultural equipment, the spatial diffusion of potential energy of advanced agricultural technology, and the extension of agricultural products before and after deep processing can provide significant spatial spillover effects.

## Further analysis

### Threshold effect test

Based on the research results of Wang et al. (2022) and Shi and Zhang (2022) [46,72], this paper introduces the two threshold variables of *SLO* and *AIG*, analyzes the relevant mechanism of agricultural technological progress to promote rural revitalization through scale and industrial agglomeration through the introduction of threshold variables.

**Fig 4:**
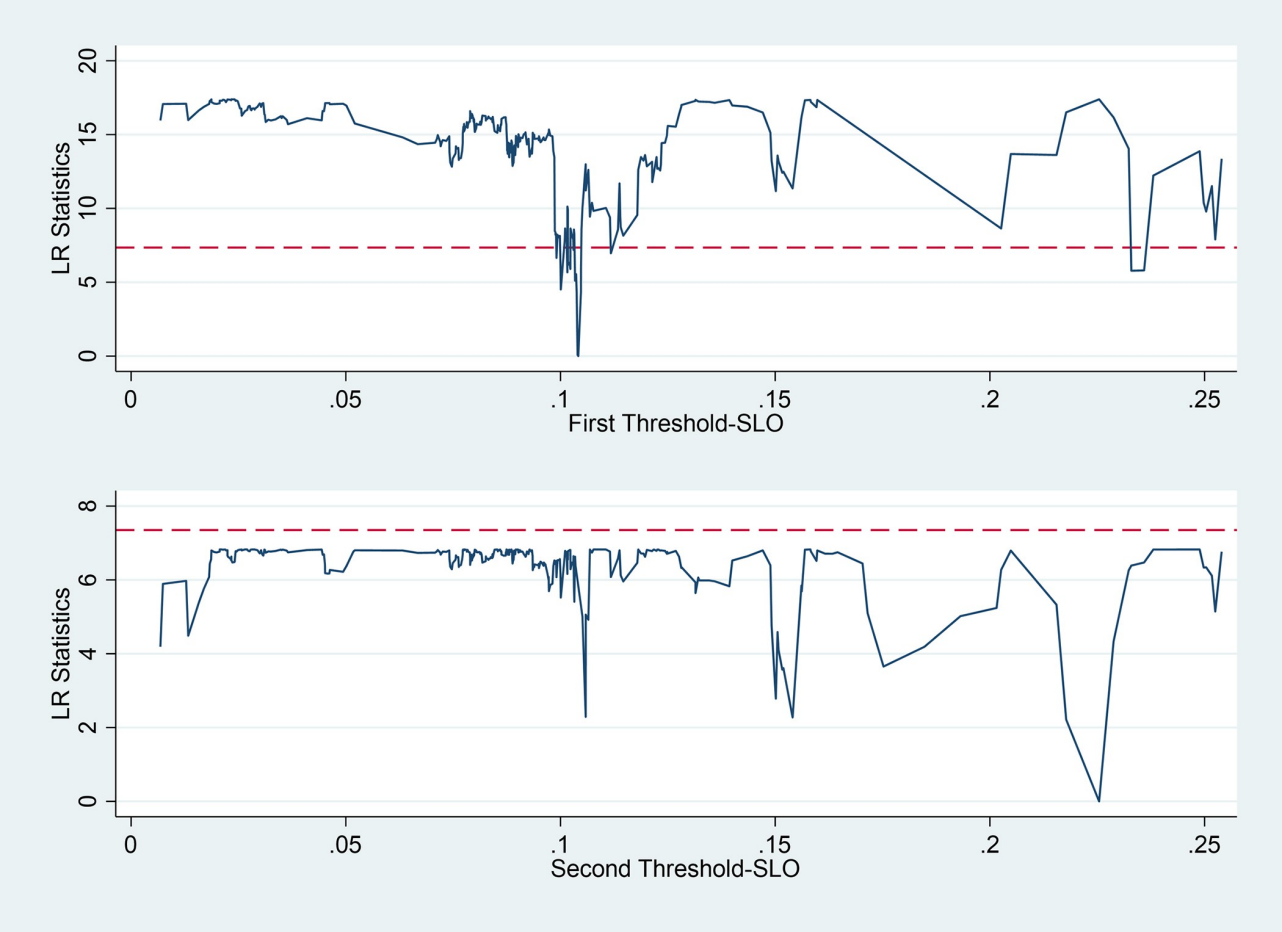
Identification of double thresholds for SLO.

In this paper, 300 samples were taken by the bootstrap method to test the significance of the single, double, and triple thresholds of *SLO* and *AIG*, as shown in Fig 4 and Table 7 below.

**Table 7:** Threshold effect significance test.

Threshold variables	Threshold type	*F-statistic*	*P statistic*	10%	5%	1%	Thresholds	95% confidence interval
*SLO*	singleness	25.30***	0.003	13.431	16.135	21.487	0.104	[0.103 0.105]
	double	6.94	0.650	17.684	21.595	29.111		
	triple	13.34	0.103	13.394	20.310	38.013		
*AIG*	singleness	17.801***	0.006	6.649	12.718	16.326	1.174	[1.148 1.253]
	double	6.532**	0.041	1.880	12.586	39.164	2.226	[2.162 2.309]
	triple	13.62	0.396	28.935	33.604	50.021		

As shown in Table 7, the threshold model test results show that the *SLO* passes the single threshold test, which verifies Hypothesis H3. The threshold estimate of the threshold variable *SLO* is 0.104, and the 95% confidence interval is (0.103 0.105), which passes the test at the significance level of 1%, and the LR statistic in the maximum likelihood ratio function graph is lower than the critical value at the significance level of 1%, which is consistent with the test results in Table 6. *AIG* passed the single threshold and double threshold tests, and the triple threshold identification failed the significance test, thus verifying Hypothesis H4. That is, the impact of agricultural technological progress on rural revitalization has a threshold effect based on the constraints of *SLO* and *AIG*.

### The threshold constraint effect based on *Land-Scale Operation*

From the threshold test results, it can be seen that when the land-scale operation (*SLO*) is at a different level, the impact of agricultural technological progress on the level of rural revitalization is different; thus, the two share structural characteristics.

As shown in Table 8, when the *SLO* level is less than the single threshold, the impact of agricultural technological progress on rural revitalization is significant at the 1% level, and the estimation coefficient is negative. When the *SLO* is greater than the single threshold, the estimation coefficient of the impact of agricultural technological progress on rural revitalization is 0.0122, which is significantly improved and passes the significance test of 5%, and research Hypothesis H3 is verified.

**Table 8:** Regression results of threshold model.

Variables and statistical parameters	Nationwide (13)	The main grain producing areas (14)	Nonmajor grain producing areas (15)
*ATA·I*(*SLO>γ*)	0.0122**	0.0197***	0.0425***
	(0.0502)	(0.0017)	(0.0063)
*ATA·I*(*SLO*≤*γ*)	-0.0337***	-0.0181***	0.0614
	(0.0022)	(0.0012)	(0.0146)
*FKL*	0.0464***	0.0696**	0.2703***
	(0.0076)	(0.0073)	(0.0358)
*FIN*	0.1762***	0.3116	0.4322**
	(0.0020)	(0.0033)	(0.0249)
*AIP*	-0.0580***	0.0401**	-0.0559**
	(0.0081)	(0.0186)	(0.0034)
*RNE*	-0.0065**	-0.0026**	-0.0094****
	(0.0116)	(0.0074)	(0.0042)
*F−stata*	94.17	71.08	52.38
*R* ^2^	0.551	0.683	0.409
*sigma_u*	0.094	0.075	0.066
*sigma_e*	0.031	0.019	0.018
*rho*	0.897	0.742	0.672

In the low-level stage of *SLO*, the benefits of *SLO* during this period are unstable, facing strong risks such as technology application, factor price increases, warehousing sales, financing constraints, etc., and it is almost impossible to provide stable income expectations and investment incentives for large-scale business entities, so its production and operation are more profit-seeking, and *SLO* has opportunistic motives and short-term income maximization tendencies. The expansion of the scale of land circulation raises the expectation of the stability of income for *SLO*, which is conducive to the long-term investment decisions of agricultural scale operators in land quality protection, application of new technologies, and production specialization (Xue et al. 2023) [74], and more importantly, the regional centralization and specialization of agricultural planting improve the decision-making ability regarding agricultural planting. More importantly, the regional concentration and specialization of agricultural planting have improved the decision-making abilities of agricultural scale operators to expand investment in agricultural machinery, enriched the choice of scattered farmer households to collectively purchase agricultural productive services, and promoted the development of agricultural productive services.

The regression results in Table 7, Columns (14) and (15) show that the effect of agricultural technological progress on rural revitalization is more significant in non-grain-producing areas under the constraints of *SLO*.

Most of China’s main grain-producing areas are in the northeast and central regions of China, where land is concentrated and contiguous, and agricultural technologies such as mechanization and chemicalization that adapt to the concentration of land scale can not only improve agricultural production efficiency but also crowd out rural labor more obviously (Luo and Zhang, 2020)[75]. The land scale of the nonmajor grain-producing areas is small, mostly in the eastern, southern, and southwestern parts of China, and natural factors such as topography, soil quality, and climate are similar. The progress of agricultural technology is biased toward agricultural diversification and characteristic agriculture, and the exchange of agricultural technology and innovation resources is convenient, which is conducive to the spatial diffusion of technology (Liang 2022) [76].

### The threshold constraint effect based on *Agricultural-Industry Agglomeration*

*AIG* does not have special requirements for land scale, but there is a certain "path dependence" effect, especially in provinces with the comparative advantage of initial agricultural natural resource endowment, the organic agglomeration of farmers and enterprises with the specialization and large-scale operation of certain agricultural products as the core promotes the networking of market organization, the expansion of production scale, and the diffusion and promotion of agricultural technology, and finally promotes the development of the rural social economy and realizes rural revitalization.

As shown in Table 9, the impact of agricultural technological progress on rural revitalization under the constraints of *AIG* shows obvious threshold characteristics.

**Table 9:** Threshold variable model regression results based on *AIG*.

Variables and statistical parameters	Nationwide (13)	The main grain producing areas (14)	Nonmajor grain producing areas (15)
$ATA(AIG\geq \gamma_{2})$	0.0054***	0.0107*	0.0228**
	(0.0344)	(0.0011)	(0.0107)
$ATA\cdot I(\gamma_{1}\leq AIG<\gamma_{2})$	0.0329***	0.0612***	0.0140**
	(0.0183)	(0.0172)	(0.0117)
$ATA\cdot I(AIG<\gamma_{1})$	-0.0127**	-0.0079***	0.0137**
	(0.0426)	(0.0014)	(0.0085)
*FKL*	0.0456***	0.0338***	0.2516***
	(0.0666)	(0.0038)	(0.0604)
*FIN*	0.0887***	0.0701	0.1308**
	(0.0401)	(0.0286)	(0.1326)
*AIP*	-0.0582**	-0.1925***	-0.0155
	(0.0562)	(0.066)	(0.2155)
*RNE*	-0.0041***	-0.0086**	-0.0033**
	(0.0047)	(0.0085)	(0.0058)
*F−stata*	94.14	88.54	79.60
*R* ^2^	0.463	0.440	0.312
*sigma_u*	0.094	0.083	0.069
*sigma_e*	0.032	0.022	0.027
*rho*	0.897	0.752	0.703

According to the results of Table 9, when the *AIG* level is less than the first threshold, agricultural technological progress under the constraints of *AIG* has an inhibitory effect on rural revitalization, which shows that *AIG* strengthens the "U" shaped relationship between agricultural technological progress and China’s interprovincial rural revitalization. Zhang et al. (2021) [53] found that there is a certain path dependence on *AIG*, that is, the path of “agglomeration-externality-spatial”, and in agglomeration areas, large-scale agricultural capital goods such as agricultural machinery, cold chain warehouses, and water conservancy equipment need to be reduced through mutual assistance and cooperation mechanisms within the industry to reduce the unit cost of use.

At the same time, the difference between the results of Table 9, Columns (14) and (15) shows that the planting of crops is severely constrained by site conditions such as the landform, soil, and hydrology, which leads to the geographical specificity of *AIG*, so even if there is spatial diffusion of agricultural capital investment, *AIG* must be limited and constrained by the natural forces of life (Luo 2000) [48], and the learning effect and spatial spillover effect of neighboring land will be greatly reduced. The crowding out effect of agricultural technology on labor exceeds the spatial synergistic effect of industrial agglomeration. To a certain extent, this also explains the spatial heterogeneity of the impact of agricultural technological progress on China’s interprovincial rural revitalization. In the main grain-producing areas, the land scale is larger, the large-scale operation of agricultural production is obvious, the fixed input of large-scale agricultural capital goods is high, and the impact of geographical location and asset specificity of *AIG* is also more obvious, which weakens the effect of spatial spillover.

## Discussion

Different from the policy logic of China’s rural revitalization, the underlying thinking of China’s rural society focuses on how to maintain their low-cost lifestyles among the left-behind population in rural families, which has become a major obstacle to rural land transfer. With the transformation of China’s rural society and the advancement of urbanization, the opportunity cost of rural residents to farm is rising, and the relative benefits of land fragmentation are declining significantly, but this does not mean that large-scale agricultural operation has become an inevitable trend in some parts of China.

1. At the low level of large-scale operation, the progress of agricultural technology has released the rural labor force, and the rural left-behind residents have increased their agricultural output through chemical agricultural farming, but this high-cost input has not reduced the production cost and improved the efficiency of agricultural operation but has become a way for the rural left-behind population to maintain their low-cost lifestyle.
2. Risks such as strong technology application, factor price increases, warehousing and sales, and financing constraints make it almost impossible to provide stable expectations and investment incentives for large-scale operators, so their production and operation are more profit-seeking, and *SLO* has opportunistic motives and short-term income maximization tendencies, so agricultural technological progress may not promote *SLO*, realize the reduction of chemical inputs in agricultural production, and improve agricultural operation efficiency.
3. Any expansion of infrastructure investment and the promotion of public technology can promote the social and economic development of rural areas and promote the process of rural revitalization. However, there is also such a risk: on the one hand, the dispersion of rural households causes the promotion and application of agricultural public technology to face high policy costs, which makes it difficult for the financial support policy to achieve effective strength and incentivize farmers to expand specialized production. On the other hand, there is a deviation in the performance consideration of the policy in practice, resulting in the rural business entities simply imitating the traditional industries at the low end of the value chain with a low technical threshold and the planting and breeding industry to capture the financial support funds for their own interests, which not only creates a situation of "sporty" governance in rural areas but also cannot effectively promote the high-level agglomeration of agricultural industry elements.
4. The implementation of China’s rural revitalization policy faces a unique socio-economic environment. Factors such as the outflow of rural labor and the conservative lifestyle of rural residents hinder the transfer of land and the promotion of large-scale operations. Although advancements in agricultural technology offer the potential to enhance productivity, they may not necessarily lead to cost reduction and efficiency improvement. This reflects rural residents’ concerns about the application of technology and market risks, especially as the risks associated with large-scale operations increase due to policy and market uncertainties. Consequently, China’s rural revitalization efforts are not solely focused on economic development; they are intricately intertwined with issues of social stability, cultural preservation, and the complexities of policy implementation.

## Conclusion and policy recommendations

The results show that the siphon effect of China’s interprovincial rural revitalization on neighboring areas cannot be ignored, while the impact of agricultural technological progress on interprovincial rural revitalization is spatially heterogeneous due to the expectation of the stability of income for *SLO* and the characteristics of the exclusivity of agricultural production geographical locations. Therefore, the article proposes the following policy recommendations:

First, agricultural technological progress is an important way to integrate the input structure of agricultural production factors such as labor and agricultural machinery and is an important driving force for promoting rural revitalization. Therefore, we should further improve the input structure of factors with the help of institutional design and policy regulation, expand the application and promotion of agricultural technology, and give full play to the role of technological progress in promoting rural revitalization. At the same time, it is necessary to optimize the spatial distribution of agricultural technology scientific research investment, promote cross-regional exchanges and cooperation of cutting-edge technologies in agriculture, actively explore the framework of interregional agricultural technology cooperation, breakdown the barriers hindering agricultural technology implementation, such as market access restrictions and local protectionism, fully grasp the positive spatial spillover effect of agricultural technological progress, and enhance the radiation capacity of rural revitalization in neighboring areas through the spillover effect and trickle-down effect of technology diffusion.

Second, land is the most basic factor of production in rural areas, and large-scale operations of farmland should be guided to revitalize land resources to empower rural revitalization. Land fragmentation is the basic national condition of China’s agriculture, the system of collective ownership of rural land has long been unclear in property rights, the separation of man and land has not been fundamentally alleviated, disputes over farmland circulation are continuous, and the enthusiasm of rural households to participate in land circulation is not high. Therefore, in promoting *SLO*, the government should improve and deepen the measures for confirming the right to land contracting and management, resolve the transaction risks in land circulation, protect the rights and interests of land contracting and operation of large-scale operators, provide stable income expectations and investment incentives for large-scale operators, weaken the opportunistic motivation of *SLO* and the tendency toward short-term benefit maximization, and breakdown the institutional obstacles to the insufficient development of *SLO*.

Third, agriculture is the foundation of the national economy, but it is a weak industry by nature, and if it simply relies on the primary natural resource endowment, it will inevitably lead to the agglomeration of agricultural industries with low technical thresholds and extensive and serious homogeneity. Therefore, it is necessary for the central and western regions to play a leading role in guiding the innovation, promotion and application of agricultural technology, but at the same time, we should try our best to avoid the performance consideration of industrial project selection in practice, eliminate the low-end imitation behavior of some business entities to arbitrage financial resources for their own development, and maximize the social benefits of financial support. In addition, the governments of the central and western regions should promote the integration of urban and rural factors by strengthening the supply of key factors, attract advanced production factors from cities to flow into leading enterprises in rural areas, use financial resources to favor key enterprises, comprehensively promote leading enterprise and rural collective economic belt projects with natural benefits to the poor, develop local agricultural characteristic industries in accordance with local conditions, and raise the level of *AIG*.

From the perspective of economic theory, China’s rural revitalization is also a transition from the stage of simple "industry regurgitation-feeding agriculture" to the stage of "endogenous development", so the rural revitalization strategy that China is implementing essentially promotes the process of modernization of China’s rural agriculture, which requires the promotion and application of agricultural technology as a tool and the rural economic community as the carrier to alleviate the structural embarrassment such as the lagging level of rural development and the weak endogenous development momentum. With the passage of time, we will find that rural revitalization is a common problem faced by China and the world, and it is a problem of how to maintain the economic vitality and endogenous development momentum of rural areas under urban civilization and industrial civilization, and it is an inevitable choice for the integrated development of urban and rural areas.
